# Cardiovascular and nervous system changes during meditation

**DOI:** 10.3389/fnhum.2015.00145

**Published:** 2015-03-18

**Authors:** Steven R. Steinhubl, Nathan E. Wineinger, Sheila Patel, Debra L. Boeldt, Geoffrey Mackellar, Valencia Porter, Jacob T. Redmond, Evan D. Muse, Laura Nicholson, Deepak Chopra, Eric J. Topol

**Affiliations:** ^1^Scripps Translational Science InstituteLa Jolla, CA, USA; ^2^The Chopra Center for WellbeingCarlsbad, CA, USA; ^3^Emotiv Research Pty Ltd.Sydney, NSW, Australia; ^4^Emotiv, Inc.San Francisco, CA, USA

**Keywords:** meditation, heart rate variability, blood pressure, wireless sensor technology, personalized medicine

## Abstract

**Background:** A number of benefits have been described for the long-term practice of meditation, yet little is known regarding the immediate neurological and cardiovascular responses to meditation. Wireless sensor technology allows, for the first time, multi-parameter and quantitative monitoring of an individual's responses during meditation. The present study examined inter-individual variations to meditation through continuous monitoring of EEG, blood pressure, heart rate and its variability (HRV) in novice and experienced meditators.

**Methods:** Participants were 20 experienced and 20 novice meditators involved in a week-long wellness retreat. Monitoring took place during meditation sessions on the first and last full days of the retreat. All participants wore a patch that continuously streamed ECG data, while half of them also wore a wireless EEG headset plus a non-invasive continuous blood pressure monitor.

**Results:** Meditation produced variable but characteristic EEG changes, significantly different from baseline, even among novice meditators on the first day. In addition, although participants were predominately normotensive, the mean arterial blood pressure fell a small (2–3 mmHg) but significant (*p* < 0.0001) amount during meditation. The effect of meditation on HRV was less clear and influenced by calculation technique and respiration. No clear relationship between EEG changes, HRV alterations, or mean blood pressure during meditation was found.

**Conclusion:** This is the first study to investigate neurological and cardiovascular responses during meditation in both novice and experienced meditators using novel, wearable, wireless devices. Meditation produced varied inter-individual physiologic responses. These results support the need for further investigation of the short- and long-term cardiovascular effects of mental calm and individualized ways to achieve it.

## Introduction

There is no doubt of a strong mind-heart connection, although it remains poorly understood. The critical nature of this connection is exemplified by stress-induced cardiomyopathy (Takutsubo cardiomyopathy), the ECG and cardiac biomarker changes with acute brain injury, and the heightened cardiovascular risk associated with depression, post-traumatic stress disorder and other mental health problems. Many of these interactions are believed to be mediated by the autonomic nervous system, which evidence suggests can be influenced through meditation (Ditto et al., 2006; Telles et al., 2013). However, greater widespread acceptance of meditation as a potentially important tool for improving population health and wellness is hampered by the limited data available defining its benefits. In addition, understanding how that response can influence cardiovascular function could help guide non-pharmacologic treatments of hypertension and other cardiovascular disorders (Burns et al., 2007; Djindjic et al., 2012; Mancia and Grassi, 2014).

While there have been a large number of studies on meditation, a 2007 Agency for Healthcare Research and Quality (AHRQ) evidence-based review classified the >800 studies as being of “… predominately poor quality…” (Ospina et al., 2007). Nonetheless, there is compelling evidence through functional MRI and EEG studies of significant changes in central nervous system (CNS) function during meditation, albeit primarily in studies limited to expert meditators (Lutz et al., 2004; Garrison et al., 2013). Other studies have explored the impact of meditation on autonomic nervous system (ANS) function predominately via changes in heart rate variability (HRV) with many but not all finding meditation to be associated with increases in HRV (Choi and Gutierrez-Osuna, 2011; Nesvold et al., 2012). While the acute effect of meditation on blood pressure has previously only been studied in a limited fashion (Barnes et al., 1999; Ditto et al., 2006) there is adequate evidence of a long-term benefit of a meditation practice to prompt the most recent hypertension treatment guidelines to give transcendental meditation a level IIB recommendation for the treatment of high blood pressure (Brook et al., 2013).

The recent availability of wireless mobile health (mHealth) devices for the continuous, non-obtrusive, passive monitoring, and transmission of numerous biometric parameters allows, for the first time, a more comprehensive and detailed evaluation of the acute neurological and cardiovascular responses to meditation. In the current study we utilized these technologies to evaluate the acute cardiovascular and nervous system responses in both experienced and novice meditators during mantra meditation to better understand the acute relationship between CNS, ANS and blood pressure changes during meditation.

## Methods

### Participants

The protocol and informed consent document were reviewed and approved by the Scripps Health Institutional Review Board. Participants in this study were a subset of 40 individuals from a total of ~200 who self-selected to participate in a week-long meditation and yoga retreat. All planned retreat participants were contacted and offered the opportunity to volunteer. There was no financial benefit offered to participants in either the cost of participating in the retreat or other compensation. After confirming qualification written informed consent was provided and signed online. The final study cohort consisted of the first 20 volunteers meeting the criteria for inclusion in the experienced meditator group and the first 20 meeting criteria as a novice meditator. Identification of experience and novice meditators was for study purposes only and had no influence on their activities during the week. Experienced meditators self-identified to having a regular meditation practice for over 3 months of = 70 min per week, whereas novice meditators self-identified as either having never seriously tried meditation, or having not meditated more than once a week during the prior 3 months. Exclusion criteria were; (1) Chronic requirement for the daily use of chronotropically active medications, (2) having an implantable pacemaker, or (3) known allergy to adhesives. Demographics of the study volunteers are in Table 1.

**Table 1:** **Demographics of study participants**.

	**Novice meditators (*N* = 20)**	**Experienced meditators (*N* = 20)**
**AGE IN YEARS**		
Mean, SD	53.5 (11.22)	48.9 (11.85)
Range	33–72	20–71
Sex (% male)	20	20
Average daily meditation practice in minutes (±SD)	0	32 (9.23)
**NUMBER OF CHRONIC MEDICATIONS**		
Number of individuals taking no medications	12	12
Number of individuals taking ≥ 1 medications	8	8
Median number of Medications	2	2

The trial was registered on clinicaltrials.gov web site; NCT01975415.

### Monitoring

All 40 meditators wore a HealthPatch by VitalConnect (http://www.vitalconnect.com/healthpatch). This device is an adhesive patch worn on the chest that monitors and wirelessly transmits a single-lead ECG, heart rate and respiratory rate to an iOS device. Using the raw ECG data heart rate variability was determined.

A total of 20 meditators, 10 experienced and 10 novices, also had their EEG's and blood pressures monitored continuously during meditation sessions. For EEG monitoring a multichannel, wireless headset, the Emotiv EPOC was used (https://emotiv.com). The EPOC uses a set of 14 sensors plus 2 references and connects wirelessly to Windows-based personal computers. For continuous, non-invasive blood pressure monitoring the Sotera ViSi Mobile system was used (http://www.visimobile.com). It is a multi-parameter vital signs monitoring system capable of continuous measurement and wireless transmission of core vital signs that utilizes pulse transition time to track blood pressure in a non-obtrusive manner.

All wireless device data was transmitted in real time to a HuNet Base Station (Huneo, Inc.) via a digital interface. There the data were compressed and sent to a secure, HIPAA-compliant cloud-based data storage. The data from the EPOC and HealthPatch devices were time-synchronized to the Internet during data acquisition whereas ViSi data were collected and time stamped on a local server that was also synchronized when later aggregated with other sensor data.

### Meditation sessions

**Figure 1:**
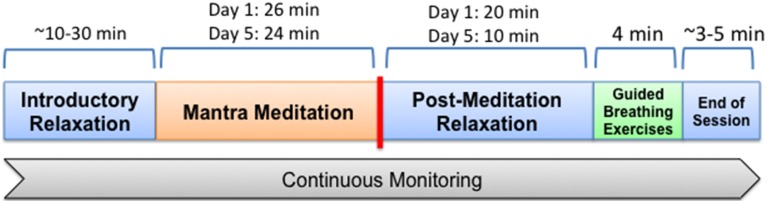
**General make up of time period in which participants were monitored**. On the first day all participated in a mantra meditation session lasting 26 min, after which they remained in place, listening to speakers for another 20 min, followed by a 4 min guided deep breathing exercise (pranayama), after which monitoring was complete. For the breathing exercise individuals were guided through cycles of inhalation for 4 s, hold for 4 s, and then exhale for 4 s via alternating nostrils. The final monitoring session was similar to the first with the meditation session lasting 24 min, followed by 10 min of quietly sitting and listening to speakers and then 4 min of guided breathing.

Volunteers were fitted with their monitoring devices on the morning of the first full day of the meditation retreat and again on the last full day. All devices were removed following the monitoring sessions with a total monitoring time of approximately 60–90 min for each of the two sessions. The general make-up of monitoring sessions is demonstrated in Figure 1.

Meditation sessions occurred in a large ballroom with all retreat participants receiving the same instructions and guidance on silent mantra meditation. All were instructed to close their eyes, relax their muscles, take a deep breath and exhale right before meditation began. No specific breathing instructions were given for the period of meditation. At the end of the meditation period a chime was rung and all were asked to release the repetition of the mantra and to then sit quietly for a few minutes before opening their eyes.

After the meditation session individuals sat quietly, listening to talks for ~10–20 min. This was followed by a 4 min guided deep breathing exercise (pranayama). For the breathing exercise individuals were guided through cycles of inhalation for 4 s, hold for 4 s, and then exhale for 4 s via alternating nostrils. After the breathing exercises monitoring was completed.

Over the ensuing 4 days between the first and last monitored sessions all participants received continued instruction and practice in meditation, yoga and other activities supporting inner calm in individual and group settings.

### Data analysis

The primary outcomes of interest were HRV as calculated from R-R intervals (the time between consecutive R waves on an ECG), continuously monitored mean arterial pressure, and EEG changes. Meditation-associated changes in the measured biometrics were compared to the post meditation period due to the consistent period of relaxed listening during that time.

#### Subjective perception of monitoring

Participants also recorded their level of distraction in relation to the number of devices worn during the meditation. Distraction level was dichotomized (distracted vs. not distracted) and a χ^2^-test was used to compare distraction between the one device group and three-device group.

#### Cardiovascular and respiratory

##### Respiratory rate

Respiratory rate was directly measured with the HealthPatch and was successfully determined in all 40 individuals on the first day but only 29 individuals on the last day. Respiratory rate was documented in breaths per minute.

##### Heart rate variability

Analyzable data for heart rate and HRV was available for 40 participants on the first day of monitoring, but on the second day data for 11 participants was either not recoverable or deemed not useful for analyses (e.g., recording abruptly concluded during the meditation session). Artifacts in RR interval data were filtered using an adaptive filter performed on the raw RR interval data from each study participant on both monitoring days (Dos Santos et al., 2013). In brief, this procedure identifies proximal and global outliers in RR interval data, simulates a more likely measurement based on proximal data points, and replaces outliers with the simulated values in an iterative process. Heart rate variability analysis was quantified on filtered RR interval data using both time-domain and frequency-domain approaches. The root mean square of successive differences (RMSSD) between adjacent RR intervals was calculated for each study participant separately for periods during the meditation session and up to 10 min immediately following meditation on both days. In this analysis, RR interval data from the first and last minute of the meditation session, and the first minute after the meditation session concluded were omitted from the analysis to eliminate the possibility of artifacts induced by study participants transitioning from the non-meditation session to meditation session, and again to a non-meditation session. A Wilcoxon signed-rank test was used to compare heart rate variability (RMSSD) between meditation and non-meditation sessions collectively, and also stratified by meditation experience on each monitoring day.

Spectral density analysis was performed using a Fast Fourier Transform (FFT) on the filtered RR interval data in 30 s segments beginning 15 min prior to the mediation session and ending 15 and 10 min after the conclusion of the meditation session on the first and second day of monitoring, respectively. Normalized high frequency (nuHF) spectral band power was derived by dividing the high frequency (0.15–0.40 Hz) spectral power by the sum of the low frequency (0.04–0.15) power plus high frequency power. Natural log high frequency (lnHF) power was also calculated. Spectral density plots were used to visualize changes associated with meditation. Similarly, a spectral density analysis was performed during the 4 min guided breathing sessions.

##### Continuous blood pressure

Data from the ViSi Mobile was either not recoverable or deemed not useful for analyses for 4 and 5 participants on the first and second monitoring day, leaving analyzable data for 16 and 15 individuals, respectively. Gross outlier measurements of mean arterial pressure were removed prior to analyses. As in the assessment of heart rate variability, mean arterial pressure was compared between the meditation session and following 15 min and 10 min non-meditation sessions on the first and second monitoring day, respectively. Therefore, mean arterial pressure was modeled as a function of meditation session using a mixed effect autoregressive model to account for the repeated, temporal dependency of the measurements. Analyses were conducted collectively among all study participants, and also stratified by meditation experience on each monitoring day.

#### Electroencephalogram

Emotiv EPOC EEG neuro-headsets were fitted to 20 volunteer subjects on each of 2 days. Due to discomfort or distraction several volunteers declined to complete the measurement, and due to contact difficulties in the fitment of headsets and the rapid evaporation of saline solvent 8 of 40 data files were incomplete and 4 were not usable. Data was bracketed into Pre-meditation, Mantra Meditation, Post Meditation relaxation, and Deep Breathing exercises. The final data set consisted of 15 complete files on the first day (9 experienced and 6 novices), with 2 cases that did not capture EEG data during the Breathing Exercise. The data set from the final day consisted of 11 complete files (4 experienced and 7 novices), with a further 3 cases missing Breathing Exercises and 3 cases missing both Post Meditation relaxation and Breathing Exercises. In total there were 10 subjects for whom complete datasets were collected over both sessions.

Real time EEG data and proprietary Emotiv “Affectiv” data were recorded to files and post analyzed. Each EEG file was band-pass filtered in the range 4–40 Hz using a 5 th order Butterworth filter, motion, and ocular artifacts were removed using automated and manual processing methods, and the remaining data divided into 2 s contiguous epochs at a 50% overlap factor. The data was windowed (Hanning filter) and passed into a FFT process using Matlab (The Mathworks, Inc. www.mathworks.com) and band power data from each sensor was produced in the theta (4–8 Hz), alpha (8–12 Hz), low beta (12–18 Hz), high beta (18–25 Hz) and gamma (25–40 Hz) bands. Individual channel data was censored according to the measured contact quality for each sensor (yellow or green indicators, corresponding to contact impedance less than approximately 80 k Ohm, significantly lower than the 1M Ohm input impedance of the EPOC amplifiers) and residual artifacts not captured by the automated processing were removed statistically using outlier analysis or by manual filtering.

Data was time synchronized to match the recorded periods of relaxed preparation (Pre-meditation), Mantra Meditation, Post-Meditation relaxation, and Breathing Exercise. Clear regions of the Pre-meditation period were selected to avoid strong motions artifacts as the subjects were entering the room and settling for the session, and also to select for periods of normal eyes-open relaxation indicated by the observation of occasional eye blink artifacts in the frontal channels.

Spectral power distributions were plotted for selected sessions in each phase using EEGLab (Delorme and Makeig, 2004). Various measures of mental activity and distribution were calculated and accumulated across subgroups of user sessions and were analyzed using SPSS to demonstrate significance of mean differences using ANOVA methods (in all cases presented *p* < 0.002 was verified). Derived features including Relative Gamma and Emotiv's “Meditation Score” were analyzed for differences between user groups and states. The Meditation Score was developed by Emotiv based on characteristic EEG signatures obtained in meditating individuals derived of the best spectral features and distribution patterns. Although gamma responses can be obscured by EMG signals (Pope et al., 2009) we chose to evaluate the Relative Gamma parameter as it has been previously shown to increase during meditation among expert meditators, but not in novice meditators (Lutz et al., 2004). It is derived from the ratio of the gamma band power to the sum of the alpha and theta band powers.

### Role of funding source

The study was funded through a grant from the Chopra Foundation. Co-investigators who are affiliated with the Chopra Center for Wellness helped design the study and recruit participants. They played no role in data acquisition or analysis but did critically review the final manuscript.

## Results

### Subjective perception of monitoring

All study participants wore their assigned monitoring devices during both sessions with the exception of two individuals who chose not to wear the EEG headset on the second day due to discomfort. Participants were asked after the second monitoring session to rank on a scale of 0–10 (0 = not at all distracted, 5 = somewhat distracted, 10 = very distracted) how distracting they found the devices. Two individuals in each device group did not record their distraction level. For those assigned to just the HealthPatch the mean score was 0.72, with 50% of the participants reporting no distraction from the single device. In comparison, among the 18 individuals assigned all 3 sensors the mean score was 2.89 with 20% reporting experiencing no distraction during the meditation (*p* < 0.05).

### Cardiovascular and respiratory changes

#### Respiratory rate

For the entire study population, over both days of monitoring, no significant changes were noted in either respiratory rate during meditation relative to the period of calm following meditation, although respiratory rate tended to be slower in both novice and experienced meditators during meditation (Table 2). When comparing experienced vs. novice meditators a statistically significant, albeit clinically small, greater decrease in respiratory rate was found among experienced meditators on the first but not last day of meditation (Table 3).

**Table 2:** **Measured biometrics during combined first and last days of meditation compared to non-meditation following it**.

	**Experienced meditators**			**Novice meditators**		
	**During meditation**	**After meditation**	***p-*value (*N*)**	**During meditation**	**After meditation**	***p*-value (N)**
Mean respiratory rate	12.8	14.8	0.074 (*N* = 20)	14.2	17.5	0.38 (*N* = 20)
Mean heart rate	69.2	69.8	0.95 (*N* = 20)	69.5	72.3	0.90 (*N* = 20)
Mean arterial blood pressure (mmHg)	99.4	101.4	0.061 (*N* = 8)	96.2	99.2	<0.001 (*N* = 8)
Heart rate variability (RMSSD)	25.6	31.2	<0.01 (*N* = 20)	32.5	33.3	0.053 (*N* = 20)
Median nuHF HRV	0.229	0.259	0.48 (*N* = 20)	0.325	0.169	<0.001 (*N* = 20)
Mean lnHF HRV	11.9	11.7	0.16 (*N* = 20)	12.4	12.0	<0.01 (*N* = 20)
Mean Scaled Meditation Score	0.400	0.287	<0.0001 (*N* = 13)	0.332	0.328	<0.01 (*N* = 13)
Mean Relative Gamma Score	0.677	0.821	<0.0001 (*N* = 13)	0.895	0.918	<0.01 (*N* = 13)

RMSSD, Root Mean Square Sequential Differences in RR intervals; nuHF HRV, normalized high frequency heart rate variability; lnHF HRV, natural log high frequency heart rate variability. N values represent number of individuals on day 1.

**Table 3:** **Differences in changes in multiple measured biometrics during meditation between experienced and novice meditators and the first and last day of the meditation retreat**.

	**First day**			**Last day**		
	**Experienced meditators**	**Novice meditators**	***p*-value (*N*)**	**Experienced meditators**	**Novice meditators**	***p*-value (*N*)**
Change in mean respiratory rate/minute	−0.72	−0.47	0.002 (*N* = 40)	−0.43	0.02	0.12 (*N* = 29)
Change in mean heart rate/minute	−0.11	−0.21	0.63 (*N* = 40)	−0.96	−0.89	0.97 (*N* = 29)
Change in mean arterial blood pressure (mmHg)	−0.50	−1.14	0.88 (*N* = 16)	0.21	0.00	0.61 (*N* = 15)
Change in heart rate variability (RMSSD)	4.95	3.07	0.36 (*N* = 40)	2.12	6.71	0.19 (*N* = 29)
Change in median nuHF HRV	0.0164	−0.0614	0.28 (*N* = 40)	0.1373	0.1263	0.78 (*N* = 29)
Change in mean lnHF HRV	−0.160	−0.417	0.12 (*N* = 40)	−0.748	−0.662	0.55 (*N* = 29)
Change in scaled meditation score	0.139	−0.015	<0.001 (*N* = 15)	0.053	0.036	<0.001 (*N* = 11)
Change in relative gamma score	−0.21	−0.003	<0.01 (*N* = 15)	0.072	−0.020	<0.01 (*N* = 11)

RMSSD, Root Mean Square Sequential Differences in RR intervals; nuHF HRV, normalized high frequency heart rate variability; lnHF HRV, natural log high frequency heart rate variability.

#### Heart rate variability

No significant change in heart rate was noted during meditation in either cohort or on either day of monitoring.

**Figure 2:**
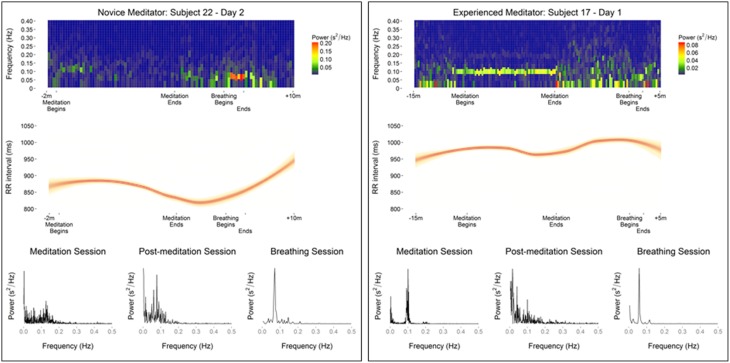
**Data from representative meditators showing differences in meditation and guided breathing-associated changes in heart rate variability as evaluated by, spectral density heat map, RR intervals, and spectral density plots**.

**Figure 3:**
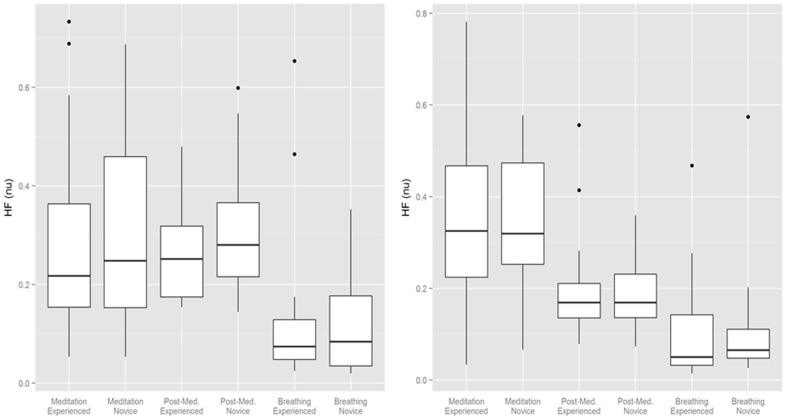
**Normalized High Frequency (NuHF) spectral power during meditation, after meditation and during guided breathing on the first and last day of monitoring**. **Bottom** and **top** of box are the first and third quartiles with the band the median with individual outliers plotted.

HRV changes in the time domain using the RMSSD demonstrated a relatively consistent decrease during the period of mantra meditation that was statistically significant in experienced meditators (*p* < 0.01) with a trend in novices (*p* = 0.053). However, when using frequency domain measurements the changes were more heterogeneous, possibly influenced by variation in respiratory rates between individuals, as demonstrated by comparisons of different analysis methods in representative individuals (Figure 2). Normalized High Frequency (nuHF) spectral power was similar in both experienced and novice meditators on the first and last day of monitoring (Table 2 and Figure 3). While on the first day there was no significant difference in nuHF between meditation and post meditation periods (*p* = 0.48), on the last day nuHF was significantly increased during meditation compared to post meditation rest (*p* < 0.001). Similar trends were observed when examining lnHF.

An exploratory analysis was carried out comparing changes in the frequency domain of HRV during guided breathing exercise compared to mantra meditation. Due to its short duration time domain analysis was not possible. In contrast to the spectral density analysis during meditation a substantial fraction of study participants showed a prominent low frequency peak at or slightly above 0.05 Hz (32 of 38 and 27 of 29 participants on the first and second day of monitoring, respectively). These differences between meditation, non-meditation quiet resting, and guided breathing are shown in representative subjects in Figure 2.

#### Continuous blood pressure

**Figure 4:**
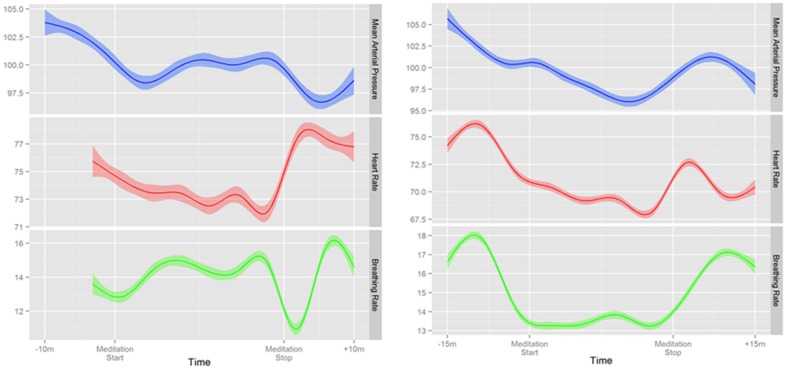
**Two representative examples of continuous changes in mean arterial pressure, heart rate, and respiratory rate before, during and after mantra meditation**.

Overall, a small but significant decrease in mean arterial pressure was found during meditation is this predominately normotensive cohort (Table 2). This was most pronounced in novice meditators with an average 3 mmHg decrease (*p* < 0.0001) and a trend of 2 mmHg (*p* = 0.061) in experienced meditators. Although blood pressure, on average, was significantly decreased with meditation there was substantial variability in response with the greatest decrease in mean arterial pressure during meditation being 16 mmHg, but also an increase of almost 22 mmHg in another individual. The time course of these changes over a meditation session and their relation to each other for two representative individuals are shown in Figure 4.

### Electroencephalogram changes

**Figure 5:**
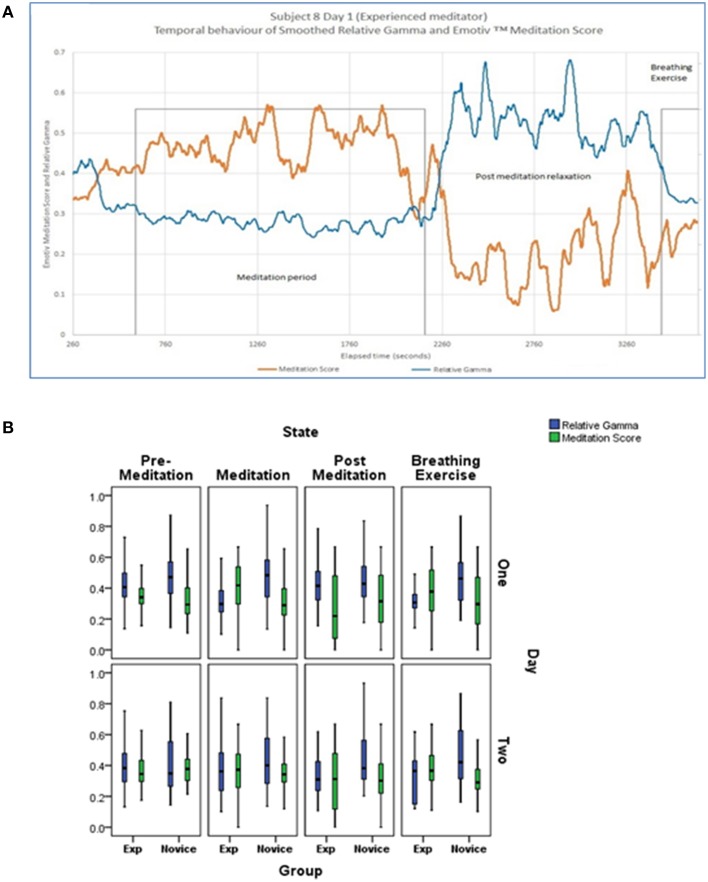
**(A)** Representative example of the temporal evolution of Relative Gamma and Emotiv™ Meditation Score for an experienced subject presented with temporal smoothing (running average over 120 s). **(B)** Cohort based changes in Relative Gamma and Meditation Score by activity and day of monitoring. Bottom and top of box are the first and third quartiles with the band the median. The difference between novice and experienced meditators at each time point and on each day was statistically significantly different at <0.05 for relative gamma and meditation score.

In order to describe global EEG changes associated with meditation we focused primarily on changes in relative gamma power and the Emotiv™ Meditation Score. A representative example of changes in these parameters over the monitoring period in an experienced meditator is shown in Figure 5 as well as the cohort-based values during the various activities on the 2 days of monitoring. Both novice and experienced meditators experienced statistically significant changes in their Meditation Score and Relative Gamma power compared to non-meditation rest (Table 2). The degree of change in both Relative Gamma and the Meditation Score was significantly greater in the experienced relative to the novice meditators (*p* = 0.04 and *p* = 0.016, respectively).

After the week of training the experienced meditators still demonstrated a numerically greater degree of EEG changes with meditation relative to novices although that difference was smaller compared to the first day and no longer statistically significant (*p* = 0.35 for relative gamma and *p* = 0.36 for Meditation Score).

Other EEG findings of note were that experienced meditators demonstrated high alpha power during all phases of the experiment including during the pre-meditation stages, whereas this was absent in novice meditators during the first day of monitoring but were significantly enhanced in the pre-meditation period in the later session. (Data not shown) Enhanced beta activity was also observed in the novice meditators from the second meditation session compared to the first.

### Relationship between parameters

**Figure 6:**
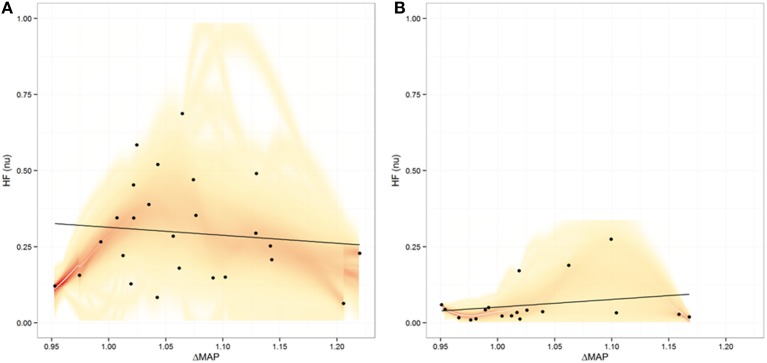
**Plot of relationship between heart rate variability expressed as normalized high frequency (nuHF) power vs. the maximal change in mean arterial pressure (MAP) during meditation (A) and during guided breathing (B)**. Shading represents the uncertainty in the best fit line

In order to evaluate for potential relationships between CNS, ANS, and cardiorespiratory changes during meditation and guided breathing exploratory analyses were carried out. No significant relationship was found between EEG changes and HRV as measured by nuHF (*p* = 0.17 and *p* = 0.16 for Relative Gamma and *p* = 0.47 and *p* = 0.50 for Meditation Score for Days one and two, respectively). Nor was there a significant relationship between breathing rate and HRV during meditation or guided breathing. On the other hand a significant association was found between the change in mean arterial blood pressure and nuHF during guided breathing (slope = 2.6844, *p* = 0.01) on the first day, but not last day (*p* = 0.40) or during meditation on either day (*p* = 0.89 and *p* = 0.07) (Figure 6).

## Discussion

While meditation has been practiced for millennia, and has been extensively studied, many uncertainties remain around the physiologic changes that occur during meditation, in particular the acute changes in non-expert and novice meditators (Ospina et al., 2007). In the current study we were able to take advantage of the relatively non-obtrusive and continuous monitoring capabilities offered through the use of multiple wireless medical devices to continuously monitor heart rate and HRV, blood pressure, respiration and EEG in novice, and experienced meditators with minimal disruption to their mantra meditation practice. In addition, we were able to track for changes in response after the novice meditators gained experience through comprehensive immersion in a meditative practice.

The uniqueness of the monitoring equipment used in this study allowed for several novel findings. This is the first study we are aware of that monitored blood pressure—continuously and unobtrusively—before, during and after meditation. Due to the disruptive nature of blood pressure cuff inflation prior studies were only able to measure blood pressure before and after meditation (Tsai et al., 2014). Using the only noninvasive FDA-approved device for continuous blood pressure monitoring we were able to confirm a small but significant drop in blood pressure during a period of meditation, notably in a predominately normotensive population. However, we were unable to identify a clear connection between meditation-related CNS or ANS changes and blood pressure changes.

As has been previously documented, we found the practice of meditation to be associated with significant changes in brain activity (Lutz et al., 2004). While prior studies have focused primarily on EEG changes in expert or experienced meditators we were able to demonstrate in first-time meditators small but significant EEG changes during their first attempt at meditation. With further practice we were able to detect a trend toward greater EEG changes with meditation in novice meditators. Contrary to a prior study of expert Tibetan monk meditators where a significant increase in Relative Gamma power was found (Lutz et al., 2004), our volunteers showed a consistent and significant decrease in this parameter during meditation. The decrease in gamma power noted in our study, in contrast to prior EEG studies in experienced meditators may be related to the fact that our volunteers were practicing a concentrative (meditating on a mantra) form of meditation, which may exhibit more of a slow oscillatory component (von Stein et al., 2009). Beyond differences in meditation focus, other potential explanations for our findings could be due to meditation artifact due to EMG interference, or related to the experience of the vastly greater experience of the monks studied previously, with 10,000 to 50,000 h of meditation experience, relative to our volunteers.

While we documented clear CNS changes with meditation, we were unable to definitively confirm ANS associated changes, at least as measured through changes in HRV. An unexpected finding from this study was that meditation was not associated with an increase in HRV as has been identified in many (Peng et al., 1999) but not all prior studies of various forms of meditation (Peng et al., 2004; Tsai et al., 2014). In fact, using the recommended method for measuring short term changes in HRV in the time domain, (Task Force of the European Society of Cardiology the North American Society of Pacing Electrophysiology, 1996) RMSDD, we found a statistically significant decrease in HRV during meditation. Interestingly, expected changes in HRV as evidenced by greater coherence was much more evident in the vast majority of meditators during guided breathing rather than during meditation (Nijjar et al., 2014), although this coherence was at low frequency, which would be anticipated when respiratory rate related. Because guided breathing was only of 4 min duration it is difficult to make valid comparisons between HRV changes during meditation relative to deep breathing, but our results do suggest that changes in HRV might be strongly influenced by respiration potentially masking more subtle changes associated with decreases in sympathetic and increases in parasympathetic tone. One measure of HRV that can help control for the influence of respiration is normalized high frequency power (nuHF) (Burr, 2007). We did identify a significant increase in nuHF but only on the final day of monitoring. This may be related to meditators being nervous on their first day of meditation, whereas by the end of the week, when all meditators were more comfortable in the group environment and with the monitoring equipment, and novice meditators had more experience, the subtle findings of the calming effects of meditation were more apparent.

There are several limitations to this study. Reflecting the non-laboratory setting of the study some participants data was not analyzable potentially influencing our power to detect more subtle changes or associations. The increased real world usability of the sensor technology utilized, in particular the EEG, brings limitations in accuracy relative to standard medical systems (Duvinage et al., 2013). The use of Emotiv's proprietary Meditation Score also unfortunately limits its reproducibility of this one measure when different EEG systems are utilized. In addition monitoring periods were relatively short, and ended rapidly after guided breathing exercises which impacted our ability to fully explore physiologic responses to respiratory changes.

## Conclusion

This is the first study to intensively monitor novice and experienced individuals during meditation for CNS, ANS, and blood pressure changes. Our results, within the limitations noted above, support that the inter-individual physiologic response to mediation by non-expert meditators is heterogeneous, as we were able to document through multiple real-time continuous metrics. Nonetheless we were able to show that meditation led to significant, measureable EEG changes even in individuals just beginning a meditation practice. Our most novel, and reliable finding however was that meditation was associated with a small, but statistically significant decrease in blood pressure in a normotensive population.

### Conflict of interest statement

SRS, Grants: Nat'l Center for Advancing Translational Sciences, Qualcomm Foundation; NEW, Grant: Nat'l Center for Advancing Translational Sciences; SP, VP (Employees), DC (Founder)–Chopra Center for Wellbeing; DLB, Scripps Dickinson Fellowship; GM, JTR, Employees, Emotiv Research, EDM, Grant: Nat'l Institute of Health; EJT, Grants: Nat'l Center for Advancing Translational Sciences, Qualcomm Foundation; Board of Directors, Dexcom, Volcano; Editor in Chief, Medscape (WebMD); Medical Advisor, AT&T.

## References

[B1] Barnes V. A. Treiber F. A. Turner J. R. Davis H. Strong W. B. (1999). Acute effects of transcendental meditation on hemodynamic functioning in middle-aged adults. Psychosom. Med. 61, 525–531. 10.1097/00006842-199907000-00017 10443761 PMC3216046

[B2] Brook R. D. Appel L. J. Rubenfire M. Ogedegbe G. Bisognano J. D. Elliott W. J.. (2013). Beyond medications and diet: alternative approaches to lowering blood pressure a scientific statement from the American heart association. Hypertension 61, 1360–1383. 10.1161/HYP.0b013e318293645f 23608661

[B3] Burns J. Sivananthan M. U. Ball S. G. Mackintosh A. F. Mary D. A. S. G. Greenwood J. P. (2007). Relationship between central sympathetic drive and magnetic resonance imaging–determined left ventricular mass in essential hypertension. Circulation 115, 1999–2005. 10.1161/CIRCULATIONAHA.106.668863 17389264 PMC3925820

[B4] Burr R. L. (2007). Interpretation of normalized spectral heart rate variability indices in sleep research: a critical review. Sleep 30, 913–919. 17682663 10.1093/sleep/30.7.913 PMC1978375

[B5] Choi J. Gutierrez-Osuna R. (2011). Removal of respiratory influences from heart rate variability in stress monitoring. Sens. J. IEEE 11, 2649–2656 10.1109/JSEN.2011.2150746

[B6] Delorme A. Makeig S. (2004). EEGLAB: an open source toolbox for analysis of single-trial EEG dynamics including independent component analysis. J. Neurosci. Methods 134, 9–21. 10.1016/j.jneumeth.2003.10.009 15102499

[B7] Ditto B. Eclache M. Goldman N. (2006). Short-term autonomic and cardiovascular effects of mindfulness body scan meditation. Ann. Behav. Med. 32, 227–234. 10.1207/s15324796abm3203_9 17107296

[B8] Djindjic N. Jovanovic J. Djindjic B. Jovanovic M. Jovanovic J. J. (2012). Associations between the occupational stress index and hypertension, type 2 diabetes mellitus, and lipid disorders in middle-aged men and women. Ann. Occup. Hyg. 56, 1051–1062. 10.1093/annhyg/mes059 22986427

[B9] Dos Santos L. Barroso J. J. Macau E. E. de Godoy M. F. (2013). Application of an automatic adaptive filter for heart rate variability analysis. Med. Eng. Phys. 35, 1778–1785. 10.1016/j.medengphy.2013.07.009 23962726

[B10] Duvinage M. Castermans T. Petieau M. Hoellinger T. Cheron G. Dutoit T. (2013). Performance of the emotiv epoc headset for P300-based applications. Biomed. Eng. Online 12:56. 10.1186/1475-925X-12-56 23800158 PMC3710229

[B11] Garrison K. A. Scheinost D. Worhunsky P. D. Elwafi H. M. Thornhill T. A. T. Thompson E.. (2013). Real-time fMRI links subjective experience with brain activity during focused attention. Neuroimage 81, 110–118. 10.1016/j.neuroimage.2013.05.030 23684866 PMC3729617

[B12] Lutz A. Greischar L. L. Rawlings N. B. Ricard M. Davidson R. J. (2004). Long-term meditators self-induce high-amplitude gamma synchrony during mental practice. Proc. Natl. Acad. Sci. U.S.A. 101, 16369–16373. 10.1073/pnas.0407401101 15534199 PMC526201

[B13] Mancia G. Grassi G. (2014). The autonomic nervous system and hypertension. Circ. Res. 114, 1804–1814. 10.1161/CIRCRESAHA.114.302524 24855203

[B14] Nesvold A. Fagerland M. W. Davanger S. Ellingsen O. Solberg E. E. Holen A.. (2012). Increased heart rate variability during nondirective meditation. Eur. J. Prev. Cardiol. 19, 773–780. 10.1177/1741826711414625 21693507

[B15] Nijjar P. S. Puppala V. K. Dickinson O. Duval S. Duprez D. Kreitzer M. J. (2014). Modification of heart rate variability: meditation versus controlled breathing alone. J. Clin. Prev. Cardiol. 3, 1–4.

[B16] Ospina M. B. Bond K. Karkhaneh M. Tjosvold L. Vandermeer B. Liang Y.. (2007). Meditation practices for health: state of the research. Evid. Rep. Technol. Assess. (Full. Rep). 155, 1–263. 17764203 PMC4780968

[B17] Peng C. K. Mietus J. E. Liu Y. Khalsa G. Douglas P. S. Benson H.. (1999). Exaggerated heart rate oscillations during two meditation techniques. Int. J. Cardiol. 70, 101–107. 10.1016/S0167-5273(99)00066-2 10454297

[B18] Peng C. K. Henry I. C. Mietus J. E. Hausdorff J. M. Khalsa G. Benson H.. (2004). Heart rate dynamics during three forms of meditation. Int. J. Cardiol. 95, 19–27. 10.1016/j.ijcard.2003.02.006 15159033

[B19] Pope K. Fitzgibbon S. Lewis T. Whitham E. Willoughby J. (2009). Relation of gamma oscillations in scalp recordings to muscular activity. Brain Topogr. 22, 13–17. 10.1007/s10548-009-0081-x 19229605

[B20] Task Force of the European Society of Cardiology the North American Society of Pacing Electrophysiology. (1996). Heart rate variability: standards of measurement, physiological interpretation, and clinical use. Circulation 93, 1043–1065. 10.1161/01.CIR.93.5.1043 8598068

[B21] Telles S. Raghavendra B. R. Naveen K. V. Manjunath N. K. Kumar S. Subramanya P. (2013). Changes in autonomic variables following two meditative states described in yoga texts. J. Altern. Complement Med. 19, 35–42. 10.1089/acm.2011.0282 22946453 PMC3546358

[B22] Tsai J.-F. Cho W. Jou S.-H. Lin C.-M. (2014). Heart rate variability and meditation with breath suspension. Biomedical Research 25, 6–10. 23621391

[B23] von Stein A. Chiang C. Konig P. (2009). Top-down processing mediated by interareal synchronization. Proc. Natl. Acad. Sci. U.S.A. 97, 14748–14753. 10.1073/pnas.97.26.14748 11121074 PMC18990

